# fMRI neurofeedback leads to long-term symptomatic reduction in treatment-resistant patients with obsessive-compulsive disorder

**DOI:** 10.1192/j.eurpsy.2023.530

**Published:** 2023-07-19

**Authors:** S. Ferreira, M. Machado-Sousa, R. Vieira, R. Magalhaes, A. Coelho, M. Picó-Pérez, P. Morgado

**Affiliations:** ^1^Life and Health Sciences Research Institute (ICVS), School of Medicine, University of Minho, Braga, Portugal; ^2^Departamento de Psicología Básica, Clínica y Psicobiología, Universitat Jaume I, Castelló de la Plana, Spain; ^3^Hospital de Braga, Braga, Portugal

## Abstract

**Introduction:**

Obsessive‐compulsive disorder (OCD) is a severe condition with a profound impact on the health, social and professional functioning of the patients. More than one third of the patients do not achieve remission of the symptoms after first‐line treatment with cognitive‐behavioral therapy and selective serotonin reuptake inhibitor medication. Neurofeedback is a promising technique that allows the non‐invasive self‐regulation of neural activity associated with symptomatic manifestation. Previous literature reported preliminary evidence of positive effects of functional magnetic resonance imaging (fMRI) neurofeedback on OCD symptoms. However, these studies have small samples and/or were not controlled. Additionally, these studies did not involve treatment‐resistant patients.

**Objectives:**

We aim at developing a fMRI neurofeedback task to treatment-resistant OCD patients and to explore the underlying brain changes.

**Methods:**

We implemented a sham‐controlled double‐blinded fMRI neurofeedback protocol to target hyperactivity in orbitofrontal regions in treatment‐resistant OCD patients with contamination/cleaning symptoms. The protocol had two sessions of neurofeedback (72 min of total training). The patients included were under treatment‐as‐usual.

**Results:**

Our preliminary results with the experimental group (*n* = 10 patients) demonstrated decreased OCD and stress symptoms three months after the neurofeedback sessions. Moreover, immediately after the neurofeedback sessions, we observed reduced functional connectivity between orbitofrontal and temporoparietal regions, and increased brain activity in dorsolateral prefrontal and premotor areas during symptomatic provocation. The brain functional changes might be associated with a better control over obsessions.

**Conclusions:**

fMRI neurofeedback led to long-term symptomatic reduction in treatment-resistant patients with OCD. Our results need further validation with the sham‐control group but highlight the efficacy of fMRI neurofeedback for refractory OCD and the necessity of prolonged neurofeedback protocols.

**Disclosure of Interest:**

None Declared

